# Letter to the editor: When do rare events become expected in HIV drug resistance?

**DOI:** 10.2807/1560-7917.ES.2026.31.16.2600334

**Published:** 2026-04-23

**Authors:** Ziad El-Khatib, Maria Wessman, Anders Sönnerborg, Jannik Fonager, Alexander Zoufaly, Gisela Leierer, Ojan Assadian, Bernhard Benka, Ndeindo Ndeikoundam Ngangro, Rami Kantor

**Affiliations:** 1 Institute for Surveillance & Infectious Disease Epidemiology, Austrian Agency for Health and Food Safety (AGES), Vienna, Austria; 2 Department of Global Public Health, Karolinska Institutet, Stockholm, Sweden; 3 Department of Infectious Disease Epidemiology and Prevention, Statens Serum Institut, Copenhagen, Denmark; 4 Department of Medicine, Karolinska Institutet, Stockholm, Sweden; 5 Section for Virus Genomics, Department of Virus & Microbiological Preparedness, Statens Serum Institut, Copenhagen, Denmark; 6 Sigmund Freud Private University, Vienna, Austria; 7 Department of Dermatology and Venereology, Medical University of Innsbruck, Innsbruck, Austria; 8 University Hospital Wiener Neustadt, Wiener Neustadt, Austria; 9 Division of Human Health, Austrian Agency for Health and Food Safety (AGES), Vienna, Austria; 10 Unit of HIV, HIV, hepatitis B/C/D, and STI, The French Public Health Agency, Saint Maurice, France; 11 Division of Infectious Diseases, Department of Medicine, Alpert Medical School, Brown University, Providence, United States

**Keywords:** HIV, transmitted drug resistance

**To the Editor**: We read with interest the report by van Kampen et.al., describing two cases of possible HIV-1 transmitted drug resistance to all currently available integrase strand transfer inhibitors (INSTIs) in the Netherlands [1]. This prompted us to consider when rare events become expected, and when they warrant action, or at least careful consideration.

Although second generation INSTIs have anchored antiretroviral therapy (ART) in the European context for years, reported transmitted INSTI resistance remains rare. Accordingly, guidelines reserve pre-ART INSTI resistance testing only for high-risk or suspected cases [2,3]. The recently reported cases in the Netherlands, although only two, raise the possibility of an evolving HIV drug resistance (HIVDR) epidemiology in the European Union/European Economic Area (EU/EEA) countries, meriting consideration for several reasons: First, they represent independent introductions of INSTI resistance into the Dutch population [1]; second, both demonstrated not only a rare event, but also predicted clinically relevant resistance to all INSTIs [1]; third, neither case would have been considered high-risk or suspected, and thus would not have met criteria for baseline INSTI resistance testing. Consequently, this resistance would likely have gone undetected in the absence of routine baseline resistance testing as part of clinical care.

Whether routine pre-ART INSTI resistance testing should be conducted remains debatable [4]. However, these two cases represent critical observations that may be interpreted as early warning European signals. We argue that the EU/EEA countries should prioritise strengthening HIVDR surveillance systems, rather than relying solely on selective testing or periodic studies, supported by the following considerations:

(i) Drug pressure is increasing globally with expanded access to and use of second generation INSTIs in all ART lines [2,3,5];

(ii) Selective testing depends on identifying individuals at risk of INSTI transmitted resistance, increasingly challenging in heterogenous epidemics, potentially missing cases [4,6];

(iii) Expanded pre-exposure prophylaxis use, including long acting cabotegravir, increases risks if used in undiagnosed HIV, resulting in suboptimal exposure, facilitating selection and potential INSTI resistance transmission, which may remain undetected;

(iv) World Health Organization (WHO) recommends HIVDR surveillance globally, although standardised approaches are primarily designed for and implemented in low- and middle-income countries (LMICs) [7]. In most EU/EEA settings, lacking routine pre-ART integrase resistance testing and limited surveillance may create a gap, potentially under-detecting INSTI resistance transmission. Notably, Sweden has implemented routine pre-treatment integrase resistance testing for several years, demonstrating feasibility and identifying pre-treatment integrase resistance (data not shown);

(v) Global health systems pressures, including reductions in HIV programme funding in LMICs, may increase HIVDR emergence risk through treatment interruptions, limited viral load monitoring and reduced resistance surveillance capacity [8]. In an interconnected epidemic, these developments may directly impact EU/EEA through migration, travel and transnational sexual networks, as well as delayed diagnosis among mobile populations. The second reported Netherland case may reflect such border dynamics [1].

Strengthening public health surveillance does not imply immediate routine pre-ART integrase resistance testing for all; current evidence indicates that it remains rare. Testing strategies must be carefully balanced against cost, clinical benefit and potential unintended consequences, including public concerns. Rather, priority should be to generate better data through strengthened and coordinated surveillance systems [5], including improving quality, timeliness and comparability across EU/EEA. This approach would enable real-time monitoring of trends and support informed, evidence-based decisions regarding if, when, and for whom routine integrase resistance testing should be implemented.

Although unreported prior ART exposure cannot be ruled out in the two cases in the Netherlands, and first generation INSTI mutations predominated in both, the question is whether such uncertainties should delay action. Given the potential consequences, we maintain that the time to act is now. What is rare today may become expected as exposure, selective pressure, and detection accumulate. While routine testing remains debatable, robust surveillance and close monitoring are essential to ensure that the rare does not become the norm.

## References

[1] van Kampen J Voermans J Soochit W Mesplède T van de Laar T van der Reijden W Two cases of possible transmitted HIV drug resistance to all currently available integrase inhibitors, the Netherlands, 2025. Euro Surveill. 2026;31(8): 2600108. 10.2807/1560-7917.ES.2026.31.8.2600108 41755723 PMC13074222

[2] European AIDS Clinical Society (EACS). Guidelines version 12.0. Oct 2023. Available from: https://www.eacsociety.org/media/guidelines-12.0.pdf

[3] Panel on Antiretroviral Guidelines for Adults and Adolescents. Guidelines for the use of antiretroviral agents in adults and adolescents with HIV. Washington DC: United States Department of Human Health and Services; 25 Sep 2025. Available from: https://clinicalinfo.hiv.gov/sites/default/files/guidelines/documents/adult-adolescent-arv/guidelines-adult-adolescent-arv.pdf

[4] Kantor R Pau AK Kozal MJ Hyle EP. Do we need routine integrase resistance testing before starting antiretroviral therapy? Lancet HIV. 2025;12(9):e664-8. 10.1016/S2352-3018(25)00108-0 40541216 PMC12265005

[5] World Health Organization (WHO). Global action plan on HIV drug resistance 2017-2021. Geneva: WHO; 1 Jul 2017. Available from: https://www.who.int/publications/i/item/978-92-4-151284-8

[6] European Centre for Disease Prevention and Control (ECDC). HIV/AIDS surveillance in Europe 2024–2023 data. Stockholm: ECDC; 28 Nov 2024. Available from: https://www.ecdc.europa.eu/en/publications-data/hiv-aids-surveillance-europe-2024-2023-data

[7] World Health Organization (WHO). Integrated drug resistance action framework for HIV, hepatitis B and C and sexually transmitted infections, 2026-2030. Geneva: WHO; 19 Nov 2025. Available from: https://www.who.int/publications/i/item/9789240117204

[8] McClure M Gandhi M. Threat of HIV and tuberculosis drug resistance after US funding cuts. Lancet Infect Dis. 2025;25(5):e256-7. 10.1016/S1473-3099(25)00209-9 40122094

